# Bioinspired Principles for Large-Scale Networked Sensor Systems: An Overview

**DOI:** 10.3390/s110404137

**Published:** 2011-04-07

**Authors:** Rune Hylsberg Jacobsen, Qi Zhang, Thomas Skjødeberg Toftegaard

**Affiliations:** Aarhus School of Engineering, Aarhus University, Ny Munkegade 120, Building 1521, 8000 Aarhus C, Denmark

**Keywords:** sensors networks, biologically inspired computing, swarm intelligence, ant colony optimization (ACO), particle swarm optimization (PSO), natural time synchronization, artificial immune system (AIS), intercellular information exchange

## Abstract

Biology has often been used as a source of inspiration in computer science and engineering. Bioinspired principles have found their way into network node design and research due to the appealing analogies between biological systems and large networks of small sensors. This paper provides an overview of bioinspired principles and methods such as swarm intelligence, natural time synchronization, artificial immune system and intercellular information exchange applicable for sensor network design. Bioinspired principles and methods are discussed in the context of routing, clustering, time synchronization, optimal node deployment, localization and security and privacy.

## 1. Introduction

The recent advances in miniaturization and low-power design have led to the development of small-sizes sensor devices that can be deployed in very large sensor networks [1]. These networks can operate unattended for years. Large-scale sensor networks will be based on wireless communication by using radio wave, inductive or capacitive coupling, light or even acoustic waves for communication [2]. Each sensor device constitutes an autonomous computing device. Individual sensors have limited computational processing and electrical power. Complex processing and the usage of a large amount of memory are not feasible. When a large amount of sensor devices are interconnected they constitute a massively distributed system [3]. Sensors are often densely deployed in an ad hoc manner and most sensors do not have a predetermined location. Furthermore, a centralized control in these networks becomes impractical.

In order to save energy, these networks have sensors that typically run with low duty cycles. Hence the amount of processing of individual nodes and the message exchange between devices are kept to a minimum. Due to these characteristics conventional methods in network design such as a unique addressing, centralized routing schemes, centralized clock distribution etc. fail to meet the constraints of massively distributed systems.

Computer scientists and engineers have often looked into nature for inspiration in search for solutions to complex problems. In biological systems there are many cases where complexity is handled by individuals with limited capabilities. By using simple rules for the behavior and the interaction among individuals a global optimum can be achieved on a large, system-level scale. Sensor networks as well as biological systems need to adapt to the varying environmental circumstances including the ability to self-organize, scale efficiently and to provide robust and resilient operation for the long-term survival of the system. These characteristics constitute the basis for the development of different approaches and algorithms at different network layer for efficient, robust and resilient communication and information networks [4,5].

By looking carefully into nature, one can observe that the dynamics of many biological systems and the laws governing them are based on a surprisingly small number of simple rules. These rules yield collaborative and effective mechanisms for resource management, task allocation, social differentiation, synchronization without the need for any central controlling element. From biological systems we find motivation for derivation of different approaches and algorithms in systems design. Bioinspired (or natural) computing [6] represents a class of algorithms focusing on the efficient computing in applications such as optimization processes and pattern recognition. A lot of research effort is put into determining the candidates among which these algorithms can bring significant advantages over conventional methods. The research and development of bioinspired sensor systems with the goals of mimicking animal senses, e.g., to perform olfactory sensing [7] or tasting [8] has caught the attention of a large number of scientists and engineers. Both the electronic tongue and the electronic nose sensor system generate an output pattern that represents a synthesis of all the components in the sample.

A few surveys of bioinspired networking and communication protocols and algorithms have been published recently [4,5]. These survey reviews the current bioinspired thinking in networking. The most well-studied areas of bioinspired network engineering are swarm intelligence [9]. In particular, the ant colony optimization (ACO) and the particle swarms optimization (PSO) algorithms have been applied to networking. Natural time synchronization, artificial immune system (AIS) [10] and intercellular information exchange [11] are methods often found as well.

In this paper we provide a brief overview of the most promising applications of bioinspired principle for distributed, networked sensor systems. In Section 2 we concentrate on the application of bioinspired methods to problems related to communications. We begin by introducing the basic bioinspired networking principles. Section 3 dwells on a number of examples that illustrate the concept of using simple principles to solve complex problems in a large-scale network. Section 4 discusses the potentials for bioinspired methods and techniques in large-scale, distributed networked systems. In Section 5, we conclude our overview.

## 2. Bioinspired Networking: A Brief Overview

Many issues in networking are formulated as multidimensional optimization problems. As network dimensions are increased both spatially and in terms of the number of nodes, centralized control of communication becomes impractical. In analogy with biological system, the individual alone is of less interest compared to the collective behavior of the system of a great number of alike individuals. This line of thinking has led to the foundation of the science and engineering discipline: swarm intelligence to describe the self-organizing properties of such system. At the same time, communication networks are subject to failure either by device and link malfunction or misuse of their capacity. From biology the immune system is known to have superior performance in the detection of malfunctioning behavior and by combining principles of immunology an AIS self-healing characteristics can be demonstrated.

### 2.1. Swarm Intelligence

The behavior of large groups of interacting small insects such as ants and bees establishes the foundation for the field of swarm intelligence. Swarm (or social) intelligence in biology has been studied intensively during the last few decades [9]. Most commonly studied are the application of ant colony optimization and particle swarm optimization algorithms (PSO).

The ant system models the way ant colonies complete difficult tasks from the cooperative behavior of a great number of individuals [12]. Ant colony optimization (ACO) is a class of optimization algorithms that is based on the observation of the collective foraging behavior of ants using stigmergic communication. This can be modeled as a distributed, self-organized system consisting of a population of simple agents interacting locally with one another and with their environment. Several papers and text books describe the ACO algorithm and its application in networking [9,12–15].

The algorithm uses stochastic techniques to search the solution space for an optimal solution. Artificial ants are the agents of search activities in the algorithm. The paths that ants travel will construct a tree when they merge into each other or reach the destination. There are two types of ants applied in the algorithm: forward ants and backwards ants. The factors that control the movement of the forward ants are the *pheromone* trails that are deposited along the edges, and the nodes’ energy which provides an estimate of how far an ant will have to travel from a node to either reach the destination. Backwards ants, *i.e.*, ants traveling back from the destination node to source nodes, perform the function of updating the information, *i.e.*, the *pheromone* levels, of their pass-by nodes. The concept of a *tabu list T_k_* is used by ants to record nodes already visited and an ant cannot travel to a node that it has already visited.

The most important aspect of ACO is the transition probability *p_ij_* for an ant to move from node *i* to node *j*. This probability represents the routing information for the exploring process. The transition probability is given by

(1) $$p_{\mathrm{ij}}=\frac{{\left(\tau_{\mathrm{ij}}\right)}^{\alpha }/d{\left(i,j\right)}^{\beta }}{\sum_{k\in J_{k}\cap T_{k}}{\left(\tau_{\mathrm{ik}}\right)}^{\alpha }/d{\left(i,k\right)}^{\beta }},$$

where the sum is over all neighboring nodes *J_k_* not on the *tabu list T_k_*, *i.e.*, *J_k_* ∩ *T_k_*. *α* and *β* are constants of the algorithm. *τ_ij_* is the *pheromone* level along the edge between node *i* and node *j*. The function d(*i, j*) is the distance between nodes *i* and *j* which is used to model the energy cost to travel between nodes. Typically the Euclidean distance is used in implementations of the algorithm. A factor *ρ* is used to describe the amount of *pheromone* that evaporates over time in accordance with expression *τ_ij_* ← (1 − *ρ*)*τ_ij_*. In addition, after completing a tour, each ant lays a quantity of *pheromone* Δ*τ_ij_*, on the edges depending on the length of the tour. The more ants that follow a particular path, the more attractive that path becomes for being followed by successive ants.

In contrast, the particle swarm optimization (PSO) algorithm models social behavior of a flock of birds e.g., searching for food [9,16–19]. It is a simple and computationally efficient non-linear optimization algorithm. The PSO algorithms is best suited with optimization problems where an optimal solution can be represented as a point on a surface in an *n*-dimensional space. *n* represents the number of optimal parameters to be determined. PSO uses a swarm of *s* candidate solutions called particles, which explore an *n*-dimensional hyperspace in search of the global solution. A particle *i* occupies position *X_id_* and has velocity *V_id_* in the *d*th dimension of the hyperspace where 1 ≤ *i* ≤ *s* and 1 ≤ *d* ≤ *n*. Each particle is evaluated through an objective function *f*(*x*_1_*, x*_2_, ..., *x_n_*), where *f* : ℝ*^n^ →* ℝ. The cost (fitness) of a particle close to the global solution is lower (higher) than that of a particle that is farther away. PSO aims to minimize (or maximize) the cost function. In the global-best version, the position where the particle *i* has its lowest cost is stored as (*pBest_id_*). In each iteration *k*, the velocity *V* and the position *X* are updated by using the expressions

(2) $$V_{\mathrm{id}}\left(k+1\right)={\mathrm{wV}}_{\mathrm{id}}\left(k\right)+\phi_{1}r_{1}\left(k\right)\left({\mathrm{pBest}}_{\mathrm{id}}-X_{\mathrm{id}}\right)+\phi_{2}r_{2}\left(k\right)\left({\mathrm{gBest}}_{d}-X_{\mathrm{id}}\right)$$

(3) $$X_{\mathrm{id}}(k+1)=X_{\mathrm{id}}(k)+V_{\mathrm{id}}(k+1),$$

where *ϕ*_1_ and *ϕ*_2_ are constants, and *r*_1_(*k*) and *r*_2_(*k*) are random numbers uniformly distributed in [0,1]. The update process is iteratively repeated until either an acceptable *gBest_d_* is achieved or a fixed number of iterations is reached. The first term in the equation uses a weight *w* to balance global and local exploration.

Essentially, PSO learns from the scenario and uses this “knowledge” to solve the optimization problem. Each “bird” is a solution, *i.e.*, a “particle” in search space. All particles have ”best” values (fitness) which are evaluated by the objective function. Particles have velocities which direct the flying of particles. The “birds”’ fly through solution space by following the “birds” with the best solutions so far.

### 2.2. Natural Time Synchronization

**Figure 1.**
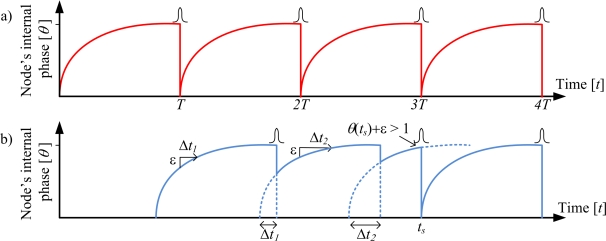
The firefly inspired time synchronization mechanism. (a) A node fires a pulse when its internal time *t* equals a multiple *n* of the oscillation period *T*. (b) An adjacent node responds to a neighbor’s pulse by incrementing its internal phase such that *θ*(*nT* + Δ*t_n_*) = *θ*(*nT*) + *ε*.

The time/clock synchronization in distributed systems is a complex issue and hard to achieve with a high degree of accuracy. In particular, server-free systems pose additional challenges for the establishment of a robust distributed clock synchronization. Recently, models for clock synchronization based on the synchronization principles of fireflies have been proposed [20–22]. The firefly synchronization mechanism is modeled as a system of pulse-coupled oscillators. These oscillators exhibit an “integrate-and-fire” behavior. The system can be described by the equation:

(4) $$\begin{array}{ccccc} \frac{{d\theta }_{i}}{\mathrm{dt}}=S_{0}-{\gamma \theta }_{i}, & & 0\leq \theta_{i}\leq 1, & & i=1,2,\ldots ,N \end{array}$$

where *θ* = *θ*(*t*) is the phase of the oscillator normalized by the oscillation period *T* and *dθ/dt* denotes the time derivative of *θ. S*_0_ and *γ* are constants. Once an oscillator reaches the phase value of one period *T*, *i.e.*, *θ_i_* = 1, it fires. Multiple oscillators are interacting by using simple pulse-coupling. When a given oscillator it pulls the other oscillators up by a fixed amount *ε* or brings them to the firing threshold as illustrated in Figure 1.

The effect of the pulse is identical to a phase shift proportional to the aggregate of pulses from oscillators firing at the same time. As a result, for almost all initial conditions the population of oscillators evolves to a state in which all the oscillators fires synchronously. As the system evolves, oscillators begin to group together in clusters that fire at the same time. This gives rise to a positive feedback process and results in a self-organized clock synchronization of the system.

### 2.3. Artificial Immune System

The artificial immune systems (AIS) mimics the property of the biological immune system. Several studies reports on the application of artificial immune systems and its application in computer science and engineering [11,23–26]. The role of the natural immune system is to protect the body from infections by continuously scanning for invading pathogens, e.g., exogenous proteins and by adapting with a proper response.

In nature, two immune responses are identified. The primary one is to launch a response to invading pathogens by provides an immediate, but non-specific, innate response. The secondary immune response remembers past encounters and, hence, it represents the immunologic memory. If pathogens successfully evade the innate response, the adaptive immune system is activated by the innate response. This secondary immune response allows a faster response the second time around showing a very specific response by using lymphocytes, which are white blood cells (T-cells and B-cells). The function of T-cells and B-cells is to recognize specific non-self antigens. B-cells respond to pathogens by producing large quantities of antibodies, which then neutralize foreign objects. In response to pathogens some T-cells produce cytokines that direct the immune response while other T-cells produce toxic granules that cause the death of pathogen infected cells.

AIS based algorithms typically exploit the immune systems characteristics of self-learning and memorization. The immune system is, in its simplest form, a cascade of detection and adaptation, culminating in a system that is remarkably effective. A simplified computational model based on the immune system has been given by Farmer, Packard and Perelson [10]. It models the set of *N* antibody concentrations *x_i_* and antigens concentrations *y_i_* in the organism by a set of coupled first order differential equations and uses a matrix of matching specificities *m_ij_* of binary classification to map parts of the antigen (the epitope) to the part of an antibody (the paratope) which recognizes an antigen. The rate at which the antibody concentration changes is given by

(5) $$\frac{{\mathrm{dx}}_{i}}{\mathrm{dt}}=c\left(\sum_{j=1}^{N}m_{\mathrm{ji}}x_{i}x_{j}-k_{1}\sum_{j=1}^{N}m_{\mathrm{ij}}x_{i}x_{j}+\sum_{j=1}^{N}m_{\mathrm{ji}}x_{i}y_{j}\right)-k_{2}x_{i},$$

where *k*_1_ and *k*_2_ are constants. The first term in the equation represents the stimulation of the paratope of an antibody of type *i* by the epitope of an antibody of type *j*. The second term represents the suppression of antibody of type *i* when its epitope is recognized by the paratope of type *j*. The constant *k*_1_ represents the possible inequality between stimulation and suppression, whereas *k*_2_ determines the rate at which cell dies. To model the full immune response we must also introduce equations to model the time-variations of antigen concentrations *y_j_*, caused by antigens generation and eliminated. To our knowledge no consistent model for antigen concentrations in AIS is found in the literature.

### 2.4. Information Exchange in Cellular Environments

Living organisms consist of billions of cells interacting with each other in a remarkably harmonic way. While current sensor network protocols suffer from scalability and efficiency issues, intercellular biological networks exhibit a robust and distributed behavior, high efficiency, and self-healing capabilities such as wound healing controlled by local cell clusters inside the organic tissues.

Signaling in biological systems occurs at multiple levels [27]. The cell communication in the human body can take place between adjacent cells (paracrine signaling) between as well as cells distributed all over the body (endocrine system). The communication between neighbor cells takes the form as an exchange of molecules (signals) via signaling pathways. One cell releases (secretes) cytokines into the intercellular space. The neighboring cell expresses a specific surface molecule, *i.e.*, the ligand (the receptor), that is activated by a change in its sterical or chemical conformation when it binds to proper cytokines. The activated receptor molecule is able to further activate intracellular molecules which enters the cell nucleus to alter gene expression. Only cells with a very specific receptor are able to receive the information, *i.e.*, the protein binds at the receptor.

For the communication over longer distances, the cells secrete hormones into blood vessels and the blood carries the molecules to all parts of the body. Signaling pathways are exposed to “noise” from interaction with ambient, residual molecules and from interference with other signaling pathways. Inhibitory pathways are interfering with the activatory pathway and the final effect is dependent on the strongest signal.

**Figure 2.**
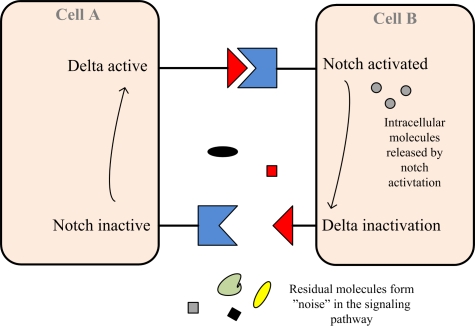
The delta-notch mechanism constitutes an effective feedback look between delta and notch ligands in neighboring cells (Adapted from [28] Figure 1).

The notch signaling pathway, which is present in most multicellular organisms, can be used to model a well-studied cell communication mechanism [28]. The notch protein sits like a trigger spanning the cell membrane, with a part of it inside and a part outside (Figure 2). Because most ligands are also trans-membrane proteins, the receptor is normally triggered only from direct cell-to-cell contact. In this way, groups of cells can organize themselves, such that, if one cell expresses a given trait, this may be switched off in neighbor cells by the intercellular notch signal. In this way, the groups of cells influence one another to distribution “tasks” in a large structure.

A simple model has been presented by Collier *et al.* [28], where *N_i_* ∈ [0*,* 1] denotes the level of notch activation, and *D_i_* ∈ [0*,* 1] denotes the level of delta activity for the *i*th cell. The following set of ordinary differential equations govern the behavior of the *i*th cell can be formulated:

(6) $$\frac{{\mathrm{dN}}_{i}}{\mathrm{dt}}=f\left({\bar{D}}_{i}\right)-N_{i}$$

(7) $$\frac{{\mathrm{dD}}_{i}}{\mathrm{dt}}=\mu \left(g\left(N_{i}\right)-D_{i}\right),$$

where *μ* is a positive constant, *D̄_i_* represents the average delta activity across the neighbors of the *i*th cell, and *f*(*D̄_i_*) and *g*(*N_i_*) are functions expressing the production rate of notch and delta activity at the cell, respectively. The rate of production of notch activity increases in response to the increasing level of delta activity in neighboring cells, whereas the rate of production of delta activity decreases with increasing level of notch activity [28].

## 3. Applying Bioinspired Networking

Distributed, networked sensor systems are not only quantitatively different, but also qualitatively different from conventional computer networks in place today. Their large scale and scope introduce new problems in design and implementation. This calls for new methods and techniques in engineering to overcome these problems. In this section the applications of bioinspired principles and methods are addressed in the context of networking. We outline the potential aspects and challenges from extrapolating these principles to distributed networked sensor systems. Table 1 summarizes the most well-established bioinspired principles and connects these to successful applications.

### 3.1. Routing

Among the many challenges encountered by sensor networks, routing issues are one of the problems that significantly block the scalability, robustness and energy-efficiency of the network. The design of routing protocols for large-scale sensor network is very difficult due to the limited available energy, the limited memory capacity and a frequent changes in network topology.

The objective of every routing algorithm is to direct traffic from sources to destinations and to maximize network performance while minimizing the cost of transmission. Routing algorithms can be broadly classified as static or dynamic. In static routers the path taken by a packet is determined on the basis of the source and destination, without regard to the current network state. This path is usually chosen as the shortest one according to some cost criterion. Adaptive (or dynamic) routers are, in principle, more attractive, because they try to adapt the routing policy to the varying traffic conditions. However, oscillations in selected paths might occur, circular routes can be generated, and large fluctuations in performances might result. Another problem with dynamic algorithms applied to, e.g., sensor networks arises when changes in the network topology occur too frequently to allow routing updates to propagate through the entire network. In the absence of global identification and unique addressing schemes of individual nodes, the network data-centric routing based on probabilistic techniques becomes attractive.

Dorigo, Maniezzo, and Colorni [12] applied ACO algorithms to the classical traveling salesman problem. The main characteristics of the model were positive feedback, distributed computation, and the use of a constructive heuristics. It was demonstrated how synergy can arise from a number of cooperative ants collaborating in search compared to the same number of ants, each one acting independently from the others.

Kassabaladis *et al.* [14] provided a survey of swarm intelligence applied to network routing. The study bases on the attracting principles such as scalability, robustness (fault tolerance), adaptation, speed, modularity, autonomy, and parallelism from the field of swarm intelligence.

Chen *et al.* [13] proposed an improved routing protocol based on the ACO algorithm for wireless sensor networks. The authors report on simulation results that show a reduced total cost of routing in a wireless sensor networks, an improved scalability, and higher efficiency. Li *et al.* [15] demonstrated an ACO-based routing protocol for wireless mesh networks. The protocol performance was compared to common ad hoc routing protocols: AODV and OLSR. The ACO-based routing protocol presented low overhead, high bandwidth utilization ratio, acceptable throughput and delay.

The above mentioned applications all represent data-centric routing schemes based on bioinspired principles. They are founded on the basis that the collective behavior of a great number of nodes can result in a scalable, robust, and energy-efficient routing network infrastructure.

### 3.2. Clustering

The grouping of nodes into clusters has been a widely pursued strategy to achieve the network scalability objective. Clustering is an important technique which aims at generating the minimum set of clusters and hereby minimizing the transmission distances in the network. The introduction of clusters allows a hierarchical routing of traffic from the sources to the destinations, conserve bandwidth and provide fault tolerant and stable operation. Furthermore, a cluster topology might be useful in networks that mix limited devices and full-functional devices deployed as cluster-heads. However, in most sensor networks applications network clustering is not desirable due to the uneven draining of resources in the network.

Several authors report on the use of PSO for network clustering: Tillett, Rao and Sahin [34] where the first to use a PSO algorithm for the clustering of nodes in a network. Dong and Qi [18] used a PSO-based clustering algorithm with an enhanced search ability. Latiff, Tsimenidis and Sharif [33] presented an energy-aware clustering for wireless sensor networks using PSO. The performance of the protocol was compared with conventional cluster-based protocols developed for wireless sensor networks, e.g., LEACH (Low-Energy Adaptive Clustering Hierarchy) and variants hereof. Guru, Halgamuge, and Fernando [32] reported on a number of extension of the PSO algorithm by introducing clustering in a wireless sensor network to reduce the total communication distance of the network and hereby decreasing the energy cost.

Charalambous and Cui [39] used bioinspired intercellular communication to achieve a compact cluster via a lateral induction model in a purely distributed and energy-efficient manner. Initially, the sensor nodes collaborate to construct a functional cluster via lateral induction followed by an lateral inhibition phase. Once clusters have been formed, a competitive scenario between nodes is created where nodes compete on which to be active or which nodes to go to sleep. Eventually, one of the active nodes becomes cluster-head as a result of competition.

Clustering has been recognized as an effective mechanism for topology control in large-scale wireless sensor networks. Inspired by the collective behavior of small animals, self-organizing clustering algorithms for large-scale networks can be developed. The algorithm does not require prior knowledge of node locations, time synchronization or other network characteristics. By using simple rules most of the nodes in a network can determine their role as either cluster head or cluster member based on purely local decisions and by using only a limited amount of broadcast messages in the network.

### 3.3. Time Synchronization

A common clock reference is essential for sensing applications for reasons such as an accurate time-stamping of sampled data. Somewhat unfortunate, time synchronization is one of the most difficult problems that an engineer of a distributed system will encounter [3].

Prior effort in time synchronization over multi-hop wireless sensor networks has mainly focused on centralized solutions using beacon signals as timing references. However, centralized solutions do not scale well and for very large-scale, distributed networks the use of a central timing references becomes impractical. Another important aspect of time synchronization is the possibility to synchronize the transmission and reception phase of nodes and hereby reducing the energy consumption by minimizing the idle listening period of the nodes. Finally, the idea is to optimize the energy-efficiency for periodic data gathering in sensor networks by a strict control of the network timing.

Bletsas and Lippman [21] demonstrated a distributed synchronization by using nearest neighbor communication. The mechanism was inspired by natural synchronization in colonies of fireflies and was implemented in an embedded wireless network. Similarly, Tyrrell and Auer [22] reported on a distributed clock synchronization. The authors pointed out that synchronization is not always obtained in a network because normal nodes are “deaf” while transmitting and hence cannot always receive synchronization messages from reference nodes.

Werner-Allen *et al.* [35] reported on the study of a decentralized time synchronization based on the Reachback Firefly algorithm. The algorithm was implemented in a sensor testbed and results were compared with simulations. In their work, the authors were able to apply and evaluate realistic effects of radio wave communication to the bioinspired synchronization mechanism and to provide a robust time-synchronized networked system.

The foreseeable scale of future networked sensor systems is encouraging scientists and engineers to use asynchronous methods, mimicking the biological systems, for the establishment of clock references. Instead of assuming that the network system is in a well-defined state at any point in time one must instead rely on a distributed system design that rely on probability.

### 3.4. Localization

Location awareness is an important property of networked sensor systems for applications such as environmental monitoring and animal tracking. Location information of the sensor nodes is used to detect and record events and to relate these events to position. Furthermore, location awareness can be used to route packets by using geometrically aware routing.

Traditionally localization techniques involve the periodic transmission of beacon signals [1]. The localization process has two phases. In the first phase, *i.e.*, the ranging phase, the nodes determine the distance between the target nodes and the neighboring beacons. In the second phase, the target nodes estimate their positions based on the ranging information. Many systems over the years have addressed the problem of automatic location sensing mainly by using techniques such as received signal strength, angle of arrival, time of arrival, and round trip time for the ranging phase.

In contrast, “beacon-free” systems rely solely on a corporative localization of nodes, where nodes work together in a peer-to-peer manner to sense and subsequent form a map of the network [40].

Gopakumar and Lillykutty [30] proposed a localization method for wireless sensor networks based on PSO. A comparison with a localization method based on simulated annealing was given. It was reported that the PSO-based method provided better localization performance compared to simulated annealing for wireless sensor network localization. Similar work was reported by Low, Nguyen and Guo [31] and a better performance of the PSO algorithm compared to conventional stochastic methods was demonstrated. Kulkarni and Venayagamoorthy [17] reported on the use of PSO and a bacterial foraging algorithms (BFA) for the distributed localization of deployed nodes in a sensor network. The two bioinspired approaches were compared. Moreover, the authors used the PSO and BFA algorithms for segmentation of terrain images for and optimal and autonomous deployment of sensor network nodes.

### 3.5. Protection (Security and Privacy)

A central challenge in computer security is the protection of the assets of a system. System assets include base resources such as processing, storage, communication and user-interface, *etc*. A key to a successful protection is the determination of the difference between normal and potentially harmful activities.

Network intrusion detection systems (IDSs) are essential elements in a computer security strategy. An IDS is a device or a software application that monitors network and system activities for malicious activities or policy violations. The IDS produce reports to a central systems that allow humans to intervene or that can be responded to by computer systems in an attempt to stop the intrusion. The analogy between the pataginic intrusion on the body and the intrusion of a malicious actor in a computer network has inspired scientists and engineers to look at intrusion detection as one of the first application of AIS [26].

A review of AIS-based IDSs has been given by Aickelin, Greensmith, and Twycross [26]. The work references a number of applications using AIS-based algorithms for IDSs. Most notable are algorithms for self-nonself detection and negative selection. Self-nonself refers to the sense of self, as in the system’s recognition of what is normal, or belonging to the system, in order to detect the opposite, that is, nonself. Negative selection refers to the process of selection of anomaly detectors based on elimination among those those that react strongly with self-antigen.

Hilker and Luther [11] introduced the concept of artificial cell communication architecture to model cell communication in organism that takes into account both the paracrine signaling and the cell signaling in the endocrine system. A middleware including artificial cell communication protocols is implemented residing between the components of the distributed system and the networking protocols. Compared to other methods the artificial cell communication has advantages in finding the receivers and the communication between a sender and several receivers.

## 4. Outlook and Future Directions

From the above discussion we have seen a broad range of applications where bioinspired principles and methods can be applied to address the problems of scalability and the computing resource scarcity of individual nodes in a networked sensor system. Bioinspired methods are best developed and analyzed in the context of a multidisciplinary conceptual framework that provides for biological models and well-founded analytical principles. The framework is based on the stages of probing the biological systems, the subsequent modeling of biological behavior—often including simplification—and algorithm development [4]. In addition, such framework shall be extended to take into account the implementation, validation and deployment aspects in order to address the many presumptions of the behavior of the large-scale network model. Subsequently we will be able to explicitly exploit the conceptual framework, in order to develop, analyze and validate novel and more sophisticated bioinspired computational schemes including those inspired by complex processes within networking.

Bioinspired models have proven to be a useful to solve complex problems in a collaborative/competitive way such as route optimization, clustering, time synchronization, localization and for system protection. We have referenced a number of examples of reports on such studies. However, many of the studies of bioinspired principles in networking are solely based on simulations and quite often also for networks of a modest size. Furthermore, much attention has been paid to the performance of the central algorithm at the expense of looking at the entire system in a realistic environment. In the future, the transformation of existing simulations into practical real-world sensor network application is desired. Inevitable, this will fuel the further development of commercial engineering products based on bioinspired principles.

A recent, very promising and largely unexplored research field of nanoscale communication networks has emerged from looking at bioinspired principles in communication. The term “nanonetwork” has been coined [41]. A nanonetwork is defined as the interconnection of multiple nanomachines using molecular communication. The propagation of communication signals in nanonetworks is different from the ordinary communication networks based on electromagnetism most notable by the fact that nanonetworks use molecules as signals. Other characteristics are a low propagation speeds and very low energy consumption required to establish communication [41]. Nanonetworks are expected to expand the capabilities of nanomachines both in terms of complexity and range of operation by allowing them to coordinate, share and exchange information. Inevitably, this will open a door to new applications in biomedicine, environmental research, industrial manufacturing, military technology, and for consumables.

## 5. Conclusions

The behavior of social animals as well as the complex biological processes in living organism are a source of inspiration in computer science and engineering. In this paper we have provided an overview of the most significant bioinspired principles for large-scale distributed networked systems such as sensor networks. We have seen how different biological systems give inspiration to different parts of network design such as routing, clustering, time synchronization, localization and protection (security and privacy). The basis for bioinspired networking has been given in order to disclose the potential and the applicability of bioinspired principles for networked sensor systems.

## Figures and Tables

**Table 1.** Applications of bioinspired principles in networking.

Biological principle	Application fields in networking	References

Swarm intelligence	Routing in computer networks, optimal node deployment, node localization, and network clustering	[12–15,17,19,29–34]
Firefly synchronization Artificial immune system	Robust and distributed clock synchronization, Misbehavior detection, intrusion detection systems, and node and rate selection	[21,22,35,36] [23,25,26,26,37]
Intercellular communication	Coordination and control in distributed systems, network clustering, and protection (security and privacy)	[11,38,39]
